# Aktueller Versorgungsstandard von Patellafrakturen in Deutschland

**DOI:** 10.1007/s00113-020-00939-8

**Published:** 2020-12-17

**Authors:** Kai Fehske, Markus T. Berninger, Lena Alm, Reinhard Hoffmann, Johannes Zellner, Clemens Kösters, Stefan Barzen, Michael J. Raschke, Kaywan Izadpanah, Elmar Herbst, Christoph Domnick, Jan Philipp Schüttrumpf, Matthias Krause, Komitee Frakturen der Deutschen Kniegesellschaft (DKG)

**Affiliations:** 1 Klinik und Poliklinik für Unfall‑, Hand‑, Plastische- und Wiederherstellungschirurgie, Universitätsklinikum Würzburg, Oberdürrbacher Straße 6, 97080 Würzburg, Deutschland; 2 Klinik und Poliklinik für Unfallchirurgie und Orthopädie, Universitätsklinikum Hamburg-Eppendorf, Hamburg, Deutschland; 3 Abteilung Unfallchirurgie, Orthopädie und Sporttraumatologie, BG Klinikum Unfallkrankenhaus Hamburg, Hamburg, Deutschland; 4 BG Unfallklinik Frankfurt am Main gGmbH, Frankfurt, Deutschland; 5 Klinik für Unfallchirurgie, Caritas-Krankenhaus St. Josef Regensburg, Regensburg, Deutschland; 6 Klinik für Orthopädie, Unfall- und Handchirurgie, Maria-Josef-Hospital Greven, Greven, Deutschland; 7 Klinik für Unfall‑, Hand und Wiederherstellungschirurgie, Universitätsklinikum Münster, Münster, Deutschland; 8 Klinik für Orthopädie und Unfallchirurgie, Department für Chirurgie, Universitätsklinikum Freiburg, Freiburg, Deutschland; 9 EUREGIO-KLINIK, Nordhorn, Deutschland; 10 Klinik für Unfallchirurgie, Universitätsklinikum Magdeburg A.ö.R., Magdeburg, Deutschland; 11 Deutsche Kniegesellschaft, Schwarzenbek, Deutschland

**Keywords:** Kniegelenk, Winkelstabile Platte, Klassische Zuggurtung, Versorgungsstrategien, Umfrage, Knee joint, Fixed-angle plate, Tension band wiring, Treatmen strategy, Survey

## Abstract

**Hintergrund:**

Die Versorgung von Patellafrakturen ist technisch anspruchsvoll. Auch wenn die radiologischen Ergebnisse zumeist zufriedenstellend sind, deckt sich dies häufig nicht mit der subjektiven Einschätzung der Patienten. Die klassische Versorgung mittels Drahtzuggurtung weist einige Komplikationen auf. Die winkelstabile Plattenosteosynthese hat sich in den letzten Jahren biomechanisch als vorteilhaft erwiesen.

**Fragestellung:**

Von wem werden Patellafrakturen in Deutschland versorgt? Wie sieht der aktuelle Versorgungsstandard aus? Haben sich „moderne“ Osteosyntheseformen durchgesetzt? Was sind die häufigsten Komplikationen?

**Material und Methoden:**

Die Mitglieder der Deutschen Gesellschaft für Orthopädie und Unfallchirurgie sowie der Deutschen Kniegesellschaft wurden aufgefordert, an einer Onlinebefragung teilzunehmen.

**Ergebnisse:**

Insgesamt wurden 511 komplett ausgefüllte Fragebogen ausgewertet. Die Befragten sind zum größten Teil auf Unfallchirurgie spezialisiert (51,5 %) und verfügen über langjährige Berufserfahrung in Traumazentren. Die Hälfte der Operateure versorgt ≤5 Patellafrakturen jährlich. In knapp 40 % der Fälle wird die präoperative Bildgebung um eine Computertomographie ergänzt. Die klassische Zuggurtung ist noch die bevorzugte Osteosyntheseform bei allen Frakturtypen (Querfraktur 52 %, Mehrfragmentfrakturen 40 %). Bei Mehrfragmentfrakturen entscheiden sich 30 % der Operateure für eine winkelstabile Plattenosteosynthese. Bei Beteiligung des kaudalen Pols dient als zusätzliche Sicherung die McLaughlin-Schlinge (60 %).

**Diskussion:**

Der Versorgungsstandard von Patellafrakturen in Deutschland entspricht weitgehend der aktualisierten S2e-Leitlinie. Nach wie vor wird die klassische Zuggurtungsosteosynthese als Verfahren der Wahl genutzt. Weitere klinische (Langzeit‑)Studien werden benötigt, um die Vorteile der winkelstabilen Plattenosteosynthese zu verifizieren.

**Zusatzmaterial online:**

In der Online-Version dieses Beitrags (10.1007/s00113-020-00939-8) finden Sie den Fragebogen, der in dieser Studie verwendet wurde. Beitrag und Zusatzmaterial stehen Ihnen auf www.springermedizin.de zur Verfügung. Bitte geben Sie dort den Beitragstitel in die Suche ein, das Zusatzmaterial finden Sie beim Beitrag unter „Ergänzende Inhalte“. 
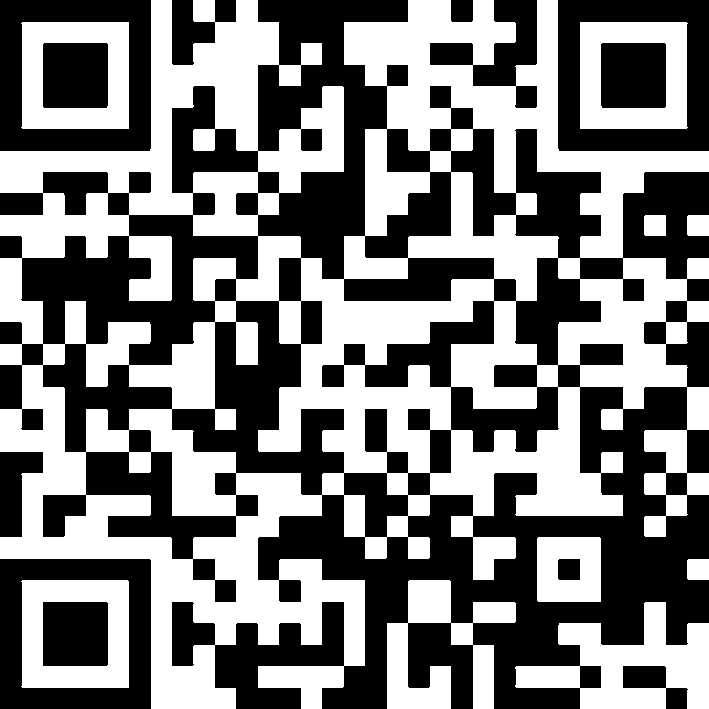

## Abstract (en)

**Background:**

The treatment of patella fractures is technically demanding. Although the radiological results are mostly satisfactory, this often does not correspond to the subjective assessment of the patients. The classical treatment with tension band wiring with K‑wires has several complications. Fixed-angle plate osteosynthesis seems to be biomechanically advantageous.

**Objective:**

Who is treating patella fractures in Germany? What is the current standard of treatment? Have modern forms of osteosynthesis become established? What are the most important complications?

**Material and methods:**

The members of the German Society for Orthopedics and Trauma Surgery and the German Knee Society were asked to participate in an online survey.

**Results:**

A total of 511 completed questionnaires were evaluated. Most of the respondents are specialized in trauma surgery (51.5%), have many years of professional experience and work in trauma centers. Of the surgeons 50% treat ≤5 patella fractures annually. In almost 40% of the cases preoperative imaging is supplemented by computed tomography. The classical tension band wiring with K‑wires is still the preferred form of osteosynthesis for all types of fractures (transverse fractures 52%, comminuted fractures 40%). In the case of comminuted fractures 30% of the surgeons choose fixed-angle plate osteosynthesis. If the inferior pole is involved a McLaughlin cerclage is used for additional protection in 60% of the cases.

**Discussion:**

The standard of care for patella fractures in Germany largely corresponds to the updated S2e guidelines. Tension band wiring is still the treatment of choice. Further (long-term) clinical studies are needed to verify the advantages of fixed-angle plates.

## Einleitung und Zielsetzung

Kniegelenknahe Frakturen gehören zu den seltenen Verletzungen des Bewegungsapparates. Aufgrund der regelhaften Beteiligung der Gelenkfläche und des damit verbundenen hohen Versorgungsanspruchs ist ihre Therapie zumeist technisch anspruchsvoll. Etwa 1 % aller Frakturen entfällt auf die Patella [2]. Mehrheitlich erleiden Männer zwischen 20 und 50 Jahren eine Patellafraktur [21], die sich zu 78,3 % im Rahmen eines Verkehrsunfalles und zu 13,7 % eines Arbeitsunfalles ereignet. Knapp 12 % der Frakturen werden im häuslichen Umfeld registriert [31]. Aufgrund der gesellschaftlichen Alterung im Rahmen der demografischen Entwicklung zeigt sich in den letzten Jahren eine Zunahme periprothetischer Patellafrakturen mit einer Inzidenz von ca. 2,5 % [5, 14]. Aber auch bei jungen Patienten können vereinzelt implantatassoziierte Frakturen, insbesondere bei der Rekonstruktion des medialen patellofemoralen Ligamentes (MPFL), beobachtet werden [1].

Die Patella ist das größte Sesambein des menschlichen Körpers; hohe Zug- und Biegekräfte beanspruchen die Osteosynthese maximal. Auch wenn es im weitaus größten Teil der Fälle zu einer vollständigen knöchernen Konsolidierung der Fraktur kommt, zeigt die Literatur lediglich in 65 % der Fälle exzellente Ergebnisse der Therapie nach einer Patellafraktur [18, 20]. Die Patienten beklagen in ca. 80 % der Fälle einen vorderen Knieschmerz, der zu starken Einschränkungen bei Alltagsaktivitäten führen kann [18]. Die Lebensqualität wird 6,5 Jahre nach dem Trauma in vielen Fällen subjektiv als stark eingeschränkt eingestuft [19]. Selbst verhältnismäßig einfache Frakturen können posttraumatisch zu einer Retropatellararthrose in bis zu 70 % der Fälle führen [29].

Ein Grund hierfür liegt in den vergleichsweise hohen postoperativen Komplikationsraten. Gerade die Zuggurtung, nach wie vor das Arbeitspferd der osteosynthetischen Versorgung bei Patellafrakturen [13, 15, 16], weist neben Materiallockerung (10 %), Fehlstellung (4,5 %), Pseudarthrose (4 %) oder Infektion (5 %) in bis zu 10 % der Fälle eine Retropatellararthrose auf, was sich in knapp 20 % in nichtzufriedenstellenden Ergebnissen widerspiegelt [23].

Aufgrund der hohen biomechanischen Belastung ist gerade bei multifragmentären Frakturen nicht immer eine Stabilisierung aller Fragmente möglich. Ein Rettungsanker ist die „Paketosteosynthese“ durch z. B. Tonnen- oder Äquatorialcerclagen. Hier kommt es neben einer Kompromittierung der Durchblutung der kleineren Fragmente insbesondere bei Beteiligung des unteren Pols auch zu technischen Problemen.

Die technischen Entwicklungen der letzten Jahre hatten somit zum Ziel, die implantatassoziierten Komplikationen zu reduzieren. Anstelle der klassischen Drahtcerclage können Kunststoffbänder aus Polyester verwendet werden, die ähnliche biomechanische Eigenschaften aufweisen [22]. Moderne Fadenmaterialien mit einem mehrsträngigen, langkettigen Kern aus Polyethylen zeigen als Zuggurtung sogar eine höhere Versagenslast als die konventionelle Drahtcerclage [33].

Eine Alternative bei einfachen Querfrakturen und Polabrissen vornehmlich des kaudalen Pols ist die kanülierte Schraubenosteosynthese, sofern die Knochensubstanz stabil genug ist, um den Schrauben Halt zu geben [7]. Als alternative Zuggurtungstechnik weisen durch kanülierte Schrauben geführte Kunststoffbänder oder Cerclagendrähte eine höhere Stabilität als die klassische Drahtzuggurtung auf [6].

Die winkelstabile Plattenosteosynthese, die ursprünglich zur Versorgung von Mehrfragment- und Trümmerfrakturen konzipiert wurde [30], stellt aktuell das „modernste“ Osteosyntheseverfahren bei Patellafrakturen dar [8]. In biomechanischen Studien konnte eine hohe mechanische Stabilität der Plattenosteosynthese gezeigt werden, die der klassischen Zuggurtungsosteosynthese signifikant überlegen war [34]. Die Weiterentwicklung der winkelstabilen Platten setzt auf kleinere Schraubendurchmesser und ist in einer Krallenplattenversion verfügbar, um insbesondere Ausrisse des kaudalen Pols besser zu adressieren. Darüber hinaus bietet sie die Option, die Kralle mit einer Schraubenosteosynthese zu kombinieren. Ein wesentlicher Vorteil der winkelstabilen Plattenosteosynthese liegt in der monokortikalen Fixationsmöglichkeit. In den wenigen bisher veröffentlichten Studien zeigen sich zufriedenstellende mittelfristige Ergebnisse: Laut Moore et al. musste die Platte bei keinem Patienten entfernt werden [24]. Dies wiederspricht allerdings den Erfahrungen einiger Autoren, wonach einige, vornehmlich jüngere Patienten, die Platte ca. ein Jahr postoperativ entfernt haben wollten [10].

Die im September 2019 aktualisierte S2e-Leitlinie zur Versorgung von Patellafrakturen der Deutschen Gesellschaft für Unfallchirurgie e. V. (DGU) gibt klare Empfehlungen u. a. zur Operationsindikation, zur Bildgebung, zum Versorgungszeitpunkt und zur Wahl des Osteosyntheseverfahrens [28]. Neben der obligaten konventionell-radiologischen Diagnostik mit Röntgenbild des Kniegelenks in 2 Ebenen sollte gemäß der Leitlinie bei multifragmentären Frakturen, Trümmerfrakturen oder Normabweichungen, wie z. B. unklaren Frakturausläufern, eine CT-Untersuchung ergänzt werden. Häufig werden jedoch das Frakturausmaß und besonders die Mitbeteiligung des oftmals mehrfragmentären, distalen Pols unterschätzt. Dies wiederum hat direkten Einfluss auf das spätere operative Vorgehen sowie das Operationsergebnis [17]. Bei erhaltener aktiver Streckhebefähigkeit des betroffenen Beines und fehlender relevanter Dislokation oder Gelenkstufe ist eine konservative Therapie indiziert. Ansonsten besteht die Notwendigkeit einer osteosynthetischen Stabilisierung mit den in der aktualisierten S2e-Leitlinie beschriebenen Standardverfahren der klassischen Zuggurtung- und/oder Schraubenosteosynthese und anterioren winkelstabilen Plattenosteosynthese. Trotz technischer und operativer Weiterentwicklungen ist die derzeitige Versorgungsrealität im deutschsprachigen Raum nicht bekannt. Das Ziel der aktuellen Studie war daher, eine Erfassung des aktuellen Versorgungsstandards von Patellafrakturen in Deutschland mittels Onlinefragebogen vorzunehmen.

## Material und Methoden

Das Komitee Frakturen der Deutschen Kniegesellschaft (DKG) hat nach einer Literaturanalyse und internen Beratung Fragen erstellt, die auf die Onlineplattform SurveyMonkey hochgeladen wurden (Zusatzmaterial online). Die Fragen befassten sich mit dem Behandlungsalgorithmus bei Patellafrakturen. Von besonderem Interesse waren u. a.:

- Von wem werden vorrangig Patellafrakturen versorgt?
- Wie werden die verschiedenen Frakturmorphologien versorgt? Gibt es bevorzugte Implantate?
- Konnte sich die winkelstabile Plattenosteosynthese aufgrund ihrer offensichtlichen biomechanischen Vorteile als Versorgungsstandard etablieren?
- Was sind die drängendsten Probleme in der Versorgung von Patellafrakturen?
- Entspricht der aktuelle Versorgungsstandard der mittlerweile aktualisierten S2e-Leitlinie?

Mit Unterstützung der Deutschen Gesellschaft für Orthopädie und Unfallchirurgie (DGOU) erfolgte über den zugehörigen E‑Mail-Verteiler eine Versendung des Fragebogens an alle Mitglieder, also auch an diejenigen, die sich nicht primär mit der Versorgung von kniegelenknahen Frakturen befassen. Insgesamt wurde die E‑Mail mit dem Aufruf zur Teilnahme an der Studie an 8439 Empfänger versendet. Die Daten wurden anonym erhoben. Eine Zuordnung zu den Befragten war nicht möglich, und nur vollständig ausgefüllte Fragebogen wurden in die Auswertung einbezogen. Der Befragungszeitraum war vom 15.04.2019 bis zum 15.06.2019.

## Ergebnisse

Insgesamt nahmen 573 Teilnehmer an der Befragung teil, was einer Teilnahmequote von 6,8 % entspricht. Hiervon konnten 511 komplett ausgefüllte Fragebogen ausgewertet werden.

Die Befragten waren fast ausschließlich in einem lokalen, regionalen oder überregionalen Traumazentrum tätig und hatten zu 51,5 % die Zusatzbezeichnung spezielle Unfallchirurgie. Bei knapp der Hälfte (49,2 %) lag die Facharzterlangung mehr als 10 Jahre zurück.

**Figure 1:**
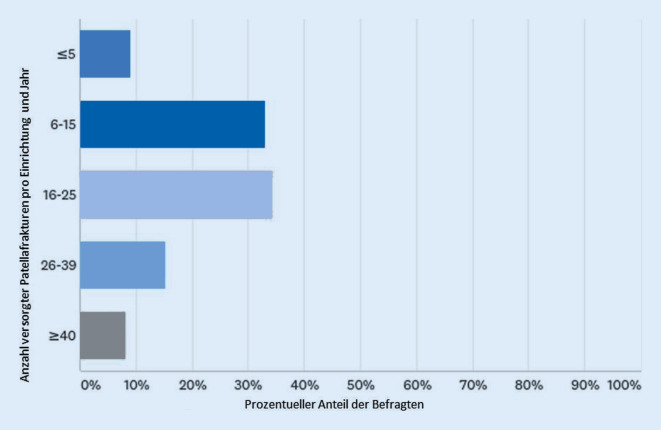

**Figure 2:**
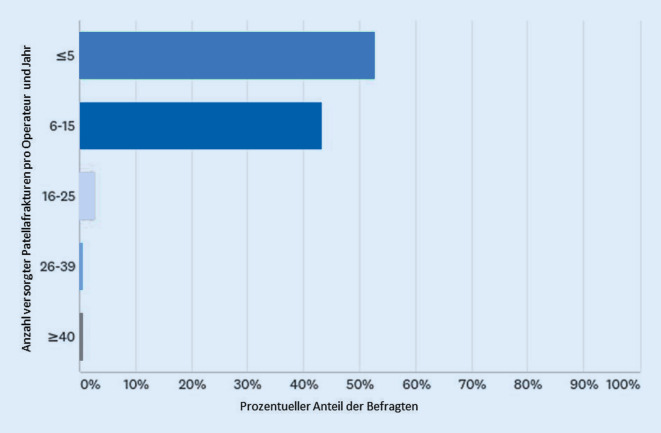

Pro Einrichtung werden jährlich im Mittel 16 bis 25 Patellafrakturen operativ versorgt (Abb. 1). Die meisten Operateure (52 %) versorgen 5 oder weniger Patellafrakturen im Jahr (Abb. 2). Die präoperative Bildgebung umfasst in allen Fällen ein konventionelles Röntgenbild; knapp 40 % der Operateure ergänzen die Diagnostik um eine Computertomographie (CT). Die Magnetresonanztomographie wird nur selten angewandt; die digitale Volumentomographie hat bisher keine Bedeutung.

Die konservative Therapie bei Patellalängsfraktur wird von 80 % der Befragten bei einem Dislokationsgrad bis zu 2 mm favorisiert.

Nach Auffassung der Befragten (51,2 %) kann die Fraktur der Patella durchaus subakut, innerhalb einer Woche posttraumatisch, ausversorgt werden. Die Antwortmöglichkeit „direkt am Unfalltag“ wird nur selten (4,6 %) als zeitliche Vorgabe angegeben.

Die klassische Zuggurtung ist nach wie vor die bevorzugte Osteosyntheseform bei allen Frakturtypen. Bei Querfrakturen entscheiden sich 52,1 % der Operateure für eine klassische Zuggurtung, in rund 17 % der Fälle ergänzt um eine zusätzliche Cerclage wie z. B. einer Tonnencerclage. Auf Rang 3 folgt die kanülierte Schraubenosteosynthese mit Cerclage (Tab. 1).

**Table 1:** 

Osteosyntheseverfahren	Anteil (%)	Anzahl (*n*)
Klassische Zuggurtung	52,05	266
Klassische Zuggurtung mit zusätzlicher Cerclage (z. B. Tonnencerclage)	17,03	87
Schraubenosteosynthese (Großfragment)	1,17	6
Schraubenosteosynthese (Kleinfragment)	5,28	27
Kanülierte Schraubenosteosynthese mit Cerclage	16,63	85
Winkelstabile Platte	6,65	34
Winkelstabile Platte mit Cerclage (z. B. Draht oder FiberTape)	0,98	5
Winkelstabile Platte mit Cerclage und zusätzlicher Schraube	0,20	1
Insgesamt	–	511

Bei der Versorgung von Mehrfragmentfrakturen wird ebenfalls die klassische Zuggurtung in Kombination mit einer zusätzlichen Cerclage von über 40 % der Befragten favorisiert. Knapp 20 % bevorzugen bei diesem Frakturtyp die winkelstabile Platte, weitere 10 % in Kombination mit einer Cerclage. Somit kommt die winkelstabile Platte in knapp 30 % bei Mehrfragmentfrakturen zur Anwendung (Tab. 2). Betrachtet man lediglich die Einrichtungen, die 16 und mehr Patellafrakturen jährlich versorgen (*n* = 302), zeigt sich ein Trend dahingehend, dass die winkelstabile Platte häufiger verwendet wird (33,4 % vs. 29,2 %).

**Table 2:** 

Osteosyntheseverfahren	Anteil (%)	Anzahl (*n*)
Klassische Zuggurtung	4,50	23
Klassische Zuggurtung mit zusätzlicher Cerclage (z. B. Tonnencerclage)	40,12	205
Klassische Zuggurtung mit zusätzlicher Schraubenosteosynthese	13,70	70
Schraubenosteosynthese	0,39	2
Kanülierte Schraubenosteosynthese mit Cerclage	10,76	55
Winkelstabile Platte	18,40	94
Winkelstabile Platte mit Cerclage (z. B. Draht oder FiberTape)	9,78	50
Winkelstabile Platte mit Cerclage und zusätzlicher Schraube	2,35	12
Insgesamt	–	511

Die McLaughlin-Schlinge wird bei einer Patellafraktur mit inferiorer Polbeteiligung als zusätzliche Sicherung der Osteosynthese von knapp 60 % der Befragten genutzt. Auch für diesen Frakturtyp werden die klassische Zuggurtung und Kombinationen mit z. B. einer additiven McLaughlin-Schlinge bevorzugt.

Intraoperativ erfolgt die Repositionskontrolle konventionell-radiologisch (91,4 %) und palpatorisch über eine Miniarthrotomie (83,2 %). Die arthroskopische Kontrolle (8,6 %) oder die Eversion der Patella mit vollständiger Exposition der Gelenkfläche (7,2 %) hat untergeordneten Stellenwert.

Als postoperative Nachbehandlung wird von 60 % eine Vollbelastung in einer Streckorthese gestattet. Die Mobilisation des Kniegelenks wird in knapp 90 % nach einem Stufenschema limitiert. Röntgenkontrollen werden direkt postoperativ und nach 6 Wochen durchgeführt. Bei komplexen Frakturen wird die postoperative radiologische Diagnostik in knapp einem Drittel um eine CT ergänzt. Nach Zuggurtungsosteosynthese kommt es nicht zu häufigeren Röntgenkontrollen.

Über 80 % der Befragten sind mit ihren Ergebnissen weitgehend zufrieden.

**Figure 3:**
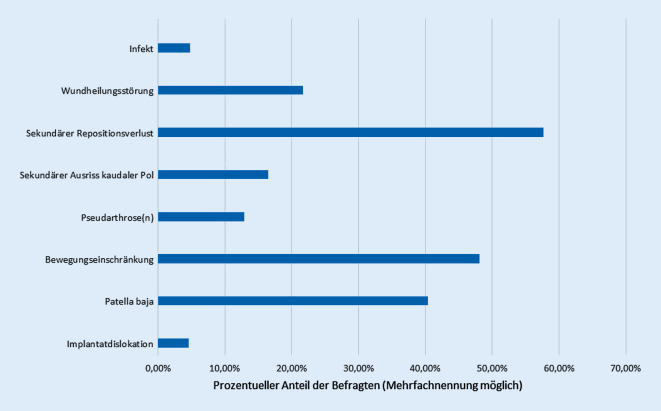

Als häufige Komplikationen oder drängendste Probleme werden neben einem sekundären Repositionsverlust (57,7 %) eine Implantatdislokation (48,1 %) und eine persistierende Bewegungseinschränkung (40,4 %) gesehen. Anhand unserer Daten können wir nicht differenzieren, ob die Komplikationsraten bestimmten Osteosyntheseformen zugeordnet werden können (Abb. 3).

## Diskussion

Die vorliegende Studie liefert erstmals umfassende Angaben zur Versorgungsrealität von Patellafrakturen in Deutschland von mehr als 500 Unfallchirurgen. Die Teilnahmequote entspricht 6,9 %. Bei vergleichbaren Onlineumfragen über den E‑Mail-Verteiler der Deutschen Gesellschaft für Orthopädie und Unfallchirurgie liegt die Rücklaufquote bei ca. 5 %. Die Versorgung von Patellafrakturen wird ganz überwiegend von erfahrenen Operateuren mit einer hohen Spezialisierung durchgeführt, was Ausdruck der häufigen Frakturkomplexität, des hohen technischen Anspruchs und der Bedeutung der Frakturlokalisation sein könnte.

Viele Antworten decken sich mit den Leitlinienempfehlungen der DGU. Die Indikation zur operativen Versorgung bei Querfrakturen mit mehr als 2 mm Dislokation, mehr als 2 mm Stufenbildung oder Aufhebung der Streckhebefähigkeit bildet die aktuelle Literaturempfehlung ab [3, 10, 11, 25, 26, 28, 32].

Die Wahl des Operationszeitpunktes entspricht ebenfalls der Leitlinie, die besagt, dass offene Frakturen notfallmäßig versorgt werden sollten und Trümmerfrakturen möglichst primär versorgt gehören. Alle anderen Frakturen können möglichst frühzeitig oder nach Abschwellen der Weichteile operiert werden [28]. Sinnvoll scheint die ebenfalls frühzeitige Versorgung bei starker Hautkontusion. Auch sollten osteochondrale Verletzungen möglichst primär versorgt werden. Anhand unserer Befragungen können wir nicht genauer nach offenen Frakturen und einem veritablen Weichteilschaden differenzieren, was sicherlich einen Einfluss auf den Versorgungszeitpunkt hat.

Das konventionelle Röntgenbild in 2 Ebenen ist der Standard zur Diagnosesicherung. In der Literatur gibt es klare Empfehlungen, zur Bestimmung der exakten Frakturmorphologie und somit auch zur operativen Planung eine CT anzuschließen. So konnten Lazaro et al. in ihrer 2013 publizierten Arbeit zeigen, dass 88 % der Patellafrakturen eine Beteiligung des distalen Pols aufwiesen, was jedoch nur in 44 % der Fälle nativ-radiologisch erkannt wurde. Konsekutiv führte die CT in 49 % der Fälle zu einer Änderung des (operativen) Behandlungsplans [17]. Zur erfolgreichen Adressierung der Frakturmorphologie und auch zur Wahl des optimalen Osteosyntheseverfahrens ist die CT somit unumgänglich. Gerade bei Verwendung winkelstabiler Platten kann die CT wichtige Aussagen zur Ausrichtung der Platte, zur Schraubenpositionierung sowie zu ggf. additiven Zugschrauben geben.

Auch wenn moderne winkelstabile Plattensysteme im Labor und in ersten klinischen Studien vorteilhaft gegenüber der klassischen Zuggurtung abgeschnitten haben, konnten sie sich im klinischen Alltag noch nicht vollständig durchsetzen. Selbst bei der Versorgung von multifragmentierten Patellae, die Hauptindikation zur Wahl einer winkelstabilen Platte, ist die klassische Zuggurtung in Kombination mit einer Äquatorialcerclage im klinischen Alltag noch das Verfahren der Wahl. Allerdings zeigt sich ein Trend, dass mit steigender Anzahl der jährlich versorgten Patellafrakturen auch die Indikation zur Plattenosteosynthese großzügiger gestellt wird.

Interessanterweise wies Labitzke bereits 1997 nach, dass das biomechanische Konzept einer exzentrischen Positionierung der (rigiden) Drahtcerclage, die bei der klassischen Zuggurtung zur Anwendung kommt, zu einer mangelhaften Kompression auf den gelenknahen Anteil der Fraktur führt [16]. Darüber hinaus konnte Zderic 2017 zeigen, dass die Zuggurtung keine Umwandlung von Zug- in Kompressionskräfte ermöglicht [33]. Die Zuggurtung als auch die Platte sind kein dynamisches, sondern ein statisches Konstrukt. Zahlreiche biomechanische Studien wiesen sowohl für die alleinige Schraubenosteosynthese, aber auch für die Kombination aus kanülierten Schrauben und Zuggurtungschlinge eine signifikant höhere Stabilität gegenüber der klassischen Zuggurtung mit Kirschner-Drähten nach [4, 8, 35]. Daher ist die derzeit persistierend hohe Anzahl an klassischen Zuggurtungsosteosynthesen sowohl bei einfachen Querfrakturen als auch bei Mehrfragmentfrakturen eine überraschende Erkenntnis aus den hier erhobenen Daten der Versorgungsrealität in Deutschland.

Der Grund für die Skepsis gegenüber der Plattenosteosynthese mag in den fehlenden Langzeitstudien, aber auch in den deutlich höheren Materialkosten im Vergleich zur klassischen Zuggurtung liegen. Neben den verbrauchten Platten muss auch das spezifische Operationssieb vorrätig sein. In den meisten Einrichtungen werden weniger als 20 Patellafrakturen jährlich versorgt, was die Amortisierung eines neu zu beschaffenden Operationssiebes erschwert, obgleich ein etwaiger Revisionseingriff bei missglückter primärer (Zuggurtungs‑)Osteosynthese diese Kosten übersteigt. Darüber hinaus könnte der individuell limitierte Erfahrungsschatz mit einer Plattenosteosynthese eine wichtige Rolle spielen. Viele Operateure wollen bewährte Operationstechniken nicht verlassen.

Die aufgeführten häufigsten Komplikation eines sekundären Repositionsverlusts, einer Implantatdislokation und einer persistierenden Bewegungseinschränkung stehen im Einklang mit den in der Literatur beschriebenen Komplikationen [9, 27, 34]. Vergleichsweise seltener werden Wundheilungsstörungen genannt. Dabei werden die peripatellären Weichteile, die allein durch den häufigen Unfallmechanismus eines direkten Anpralls verletzt sein können (z. B. Abschürfungen, Einblutung etc.), durch die operative Stabilisierung weiter belastet. Zum einen kann es durch die offene Reposition über den empfohlenen medianen oder lateral parapatellar angelegten Zugang aufgrund des dünnen Weichteilmantels zu Wundheilungsstörungen kommen; zum anderen führen die eingebrachten osteosynthetischen Materialen zu einer nichtunerheblichen Belastung der Weichteile von innen. Dies kann z. B. durch prominente Osteosynthesenanteile (Schraubenköpfe, Plattenecken) oder auch durch eine potenzielle Migration von Drähten zusätzlich verstärkt werden [18, 29].

Eine von Garcia et al. publizierte Arbeit zur Versorgungsrealität u. a. für Patellafrakturen in Deutschland konnte zeigen, dass nicht mehr Männer am häufigsten operiert werden, sondern Frauen über 50 Jahre mit einem Altersgipfel zwischen 75 und 85 Jahren [12]. Dieser alterstraumatologische Aspekt ist aus unserer Sicht ein zusätzlicher Pluspunkt für die Verwendung einer winkelstabilen Plattenosteosynthese.

## Schlussfolgerung

Die Versorgung von Patellafrakturen stellt nach wie vor einen technisch anspruchsvollen Eingriff dar. Der Versorgungsstandard in Deutschland entspricht derzeit weitgehend den Empfehlungen der aktualisierten S2e-Leitlinie der Deutschen Gesellschaft für Unfallchirurgie. Auch wenn biomechanische Studien Vorteile zugunsten winkelstabiler Plattensysteme zeigen konnten, ist nach wie vor die klassische Zuggurtung bei allen Frakturmorphologien das Osteosyntheseverfahren der Wahl. Neben fehlenden klinischen Studien mag dies auch in den deutlich höheren Implantatkosten der Platte im Vergleich zur Zuggurtung liegen. Weitere klinische (Langzeit‑)Studien werden benötigt, um zu verifizieren, ob die biomechanischen Vorteile der winkelstabilen Plattenosteosynthese zu verbesserten klinischen Ergebnissen und somit auch zum vermehrten klinischen Einsatz führen.

## Fazit für die Praxis

- Die Versorgung von Patellafrakturen ist technisch anspruchsvoll und erfolgt zumeist in Schwerpunktzentren.
- Eine Computertomographie ist für die Operationsplanung absolut empfehlenswert.
- Nach wie vor kommt die Zuggurtungsosteosynthese mit Kirschner-Drähten am häufigsten zum Einsatz.
- Moderne winkelstabile Plattensysteme zeigen gerade im Hinblick auf mehrfragmentierte Patellae biomechanische Vorteile.
- Der Versorgungsstandard in Deutschland entspricht weitgehend der aktualisierten S2e-Leitlinie der Deutschen Gesellschaft für Unfallchirurgie.

## Supplementary Information




