# Supplementary material for: Current treatment standard for patella fractures in Germany
Source: Unfallchirurg. 2020 Dec 17;124(10):832–8. [Article in German] doi: 10.1007/s00113-020-00939-8 (PMC8460507; doi:10.1007/s00113-020-00939-8)
Supplement: Supplementary file 1 [file 113_2020_939_MOESM1_ESM.pdf]

Fragebogen Behandlungsalgorithmus bei Patellafrakturen

1.) Welcher Versorgungskategorie gehören Sie an?

a) Lokales Traumazentrum
b) Regionales Traumazentrum
c) Überregionales Traumazentrum
d) Wir sind keinem Netzwerk angeschlossen

2.) In welchen Ausbildungsstand befinden Sie sich?

a) Weiterbildung zum Facharzt für Orthopädie und Unfallchirurgie
b) Weiterbildung zur Zusatzbezeichnung spezielle Unfallchirurgie
c) Weiterbildung zur Zusatzbezeichnung spezielle orthopädische Chirurgie
d) Erworbene Zusatzbezeichnung spezielle Unfallchirurgie
e) Erworbene Zusatzbezeichnung spezielle orthopädische Chirurgie

3.) Über wieviel operative Berufserfahrung verfügen Sie (seit Erreichen des FA O/U oder FA C mit Schwerpunkt Unfallchirurgie)?

a) <5 Jahre
b) 5-10 Jahre
c) >10 Jahre

4.) Wie viele Patellafrakturen versorgen Sie persönlich pro Jahr?

a) ≤5
b) 6-15
c) 16-25
d) 26-39
e) ≥40

5.) Wie viele Patellafrakturen versorgen Sie in ihrem Krankenhaus pro Jahr?

a) ≤5
b) 6-15
c) 16-25
d) 26-39
e) ≥40

6.) Welche bildgebende Diagnostik führen Sie als Standard präoperativ durch? (Mehrfachnennung möglich)

a) Konventionelles Röntgenbild in 2 Ebenen
b) Konventionelles Röntgenbild in 2 Ebenen und Patella axial
c) Computertomographie (CT)
d) Magnetresonanztomographie (MRT)
e) Digitale Volumentomographie (DVT) oder CT

7.) Bis zu welchem Dislokationsgrad führen Sie eine konservative Therapie einer Patella-Längsfraktur ohne relevante Komorbiditäten durch (intakte Retinakula)?

a) Nie
b) 2mm
c) 5mm
d) 10mm
e) >10mm

8.) Wie behandeln Sie wenig dislozierte, stabile (BV-Kontrolle) Patellafrakturen (inkl. multifragmentärer Frakturmuster) ohne relevante Komorbiditäten (intakte Retinakula)?

a) Immer operativ
b) Stabil bis 30° Flexion: konservativ (jüngerer Patient)
c) Stabil bis 60° Flexion: konservativ (jüngerer Patient)
d) Stabil bis 30° Flexion: konservativ (älterer Patient)
e) Stabil bis 60° Flexion: konservativ (älterer Patient)
f) In Abhängigkeit vom Knorpelschaden (MRT-Befund)
g) Individuell

9.) Was ist Ihre zeitliche Vorgabe bis zur operativen Versorgung einer isolierten Patellafraktur ohne limitierenden Weichteilschaden und ohne Mehrfachverletzung?

a) am Unfalltag
b) innerhalb von 48 h
c) innerhalb 1 Woche
d) nach mehr als 1 Woche

10.) Wie versorgen Sie bevorzugt einfache Querfrakturen der Patella?

a) Klassische Zuggurtung
b) Klassische Zuggurtung mit zusätzlicher Cerclage (z.B. Tonnencerclage)
c) Schraubenosteosynthese (Großfragment)
d) Schraubenosteosynthese (Kleinfragment)
e) Kanülierte Schraubenosteosynthese mit Cerclage
f) Winkelstabile Platte
g) Winkelstabile Platte mit Cerclage (z.B. Draht oder Fiber Tape)
h) Winkelstabile Platte mit Cerclage und zusätzlicher Schraube

11.) Wie versorgen Sie bevorzugt mehrfragmentierte Frakturen der Patella?

a) Klassische Zuggurtung
b) Klassische Zuggurtung mit zusätzlicher Cerclage (z.B. Tonnencerclage)
c) Klassische Zuggurtung mit zusätzlicher Schraubenosteosynthese
d) Schraubenosteosynthese
e) Kanülierte Schraubenosteosynthese mit Cerclage
f) Winkelstabile Platte
g) Winkelstabile Platte mit Cerclage (z.B. Draht oder Fiber Tape)
h) Winkelstabile Platte mit Cerclage und zusätzlicher Schraube

12.) Wie versorgen Sie Patellafrakturen mit inferiorer Polbeteiligung

a) Klassische Zuggurtung
b) Plattenostosynthese mit Haltenähten an die Platte
c) Plattenosteosynthse mit „Krallen“ für den distalen Pol
d) McLaughlin-Schlinge zusätzlich zur Osteosynthese
e) Materialkombination aus Schrauben, Drähten und Cerclage
f) Sonstige
g) der inferioren Polfraktur messe ich keine besondere Bedeutung bei

13.) Wie erfolgt die <u>intraoperative</u> Repositionskontrolle (Mehrfachnennung möglich)?

a) Palpatorisch
b) Konventionell-radiologisch (Durchleuchtung)
c) Schichtbildgebung (3D-Fluoroskopie)
d) Arthroskopisch
e) Visuelle Kontrolle ohne vollständige Exposition der Gelenkfläche
f) Eversion der Patella und vollständige Exposition der Gelenkfläche

14.) Welchen Zugang wählen Sie zur Gelenkarthrotomie?

a) Keine Arthrotomie
b) Medial
c) Lateral
d) Abhängig von Zerreißung der Retinakula

15.) Wie geben Sie die postoperative Belastbarkeit vor?

a) Entlastung
b) Teilbelastung (z.B. 20 kg)
c) Vollbelastung in einer Streckorthese (z.B. Mecronschiene)
d) Vollbelastung

16.) Wie geben Sie die postoperative Beweglichkeit vor?

a) Frei
b) Limitiert
c) Stufenschema: z.B. 2 Wochen Mecronschiene, 2 Wochen 0-0-60°, 2 Wochen 0-0-90°

17.) Welche Bildgebung führen Sie postoperativ bei einfachen Frakturen durch?

a) Konventionelles Röntgenbild in 2 Ebenen
b) Konventionelles Röntgenbild in 2 Ebenen und Patella axial
c) Computertomographie (CT)
d) Magnetresonanztomographie (MRT)
e) Digitale Volumentomographie (DVT)

18.) Welche Bildgebung führen Sie postoperativ bei komplexen Frakturen durch?

a) Konventionelles Röntgenbild in 2 Ebenen
b) Konventionelles Röntgenbild in 2 Ebenen und Patella axial
c) Computertomographie (CT)
d) Magnetresonanztomographie (MRT)
e) Digitale Volumentomographie (DVT)

19.) Wann führen Sie standardmäßig die weitere postoperative bildgebende Kontrolle nach Zuggurtung durch?

a) 2 Wochen
b) 4 Wochen
c) 6 Wochen
d) mache keine Zuggurtung

20.) Wann führen Sie standardmäßig die weitere postoperative bildgebende Kontrolle nach kanülierter Schraubenosteosynthese mit Cerclage durch?

a) 2Wochen
b) 4 Wochen
c) 6 Wochen
d) mache keine kanülierte Schraubenosteosynthese mit Cerclage

21.) Wann führen Sie standardmäßig die weitere postoperative bildgebende Kontrolle nach Plattenosteosynthese durch?

a) 2 Wochen
b) 4 Wochen
c) 6 Wochen
d) mache keine Plattenosteosynthese an der Patella

20.) Röntgen Sie nach Zuggurtungsosteosynthesen der Patella postoperativ häufiger im Vergleich zu anderen Verfahren?

a) ja
b) nein
c) mache keine Zuggurtungsosteosynthese

21.) Sind Sie mit Ihren Resultaten zufrieden?

a) Ja, uneingeschränkt.
b) Ja, weitestgehend.
c) Nein.

22.) Welche Komplikationen sehen Sie häufig? Oder: Was sind Ihrer Meinung nach die drängendsten Probleme nach operativer Versorgung einer Patellafraktur (Mehrfachnennung möglich)

a) Infekt
b) Sekundärer Repositionsverlust
c) Sekundärer Ausriß distaler Pol
d) Pseudarthrosen
e) Implantatdislokation
f) Bewegungseinschränkung
g) Patella baja
